# Bioinformatics-Based Identification of HDAC Inhibitors as Potential Drugs to Target EGFR Wild-Type Non-Small-Cell Lung Cancer

**DOI:** 10.3389/fonc.2021.620154

**Published:** 2021-03-08

**Authors:** Yizhe Wang, Chunlei Zheng, Wenqing Lu, Duo Wang, Yang Cheng, Yang Chen, Kezuo Hou, Jianfei Qi, Yunpeng Liu, Xiaofang Che, Xuejun Hu

**Affiliations:** ^1^ Department of Respiratory and Infectious Disease of Geriatrics, The First Hospital of China Medical University, Shenyang, China; ^2^ Department of Medical Oncology, The First Hospital of China Medical University, Shenyang, China; ^3^ Key Laboratory of Anticancer Drugs and Biotherapy of Liaoning Province, The First Hospital of China Medical University, Shenyang, China; ^4^ Liaoning Province Clinical Research Center for Cancer, Shenyang, China; ^5^ Marlene and Stewart Greenebaum Comprehensive Cancer Center, University of Maryland, Baltimore, Baltimore, MD, United States

**Keywords:** non-small cell lung cancer, histone deacetylase inhibitor, WGCNA, EGFR wild type, metastasis, proliferation

## Abstract

Patients with EGFR-mutant non-small-cell lung cancer (NSCLC) greatly benefit from EGFR-tyrosine kinase inhibitors (EGFR-TKIs) while the prognosis of patients who lack EGFR-sensitive mutations (EGFR wild type, EGFR-WT) remains poor due to a lack of effective therapeutic strategies. There is an urgent need to explore the key genes that affect the prognosis and develop potentially effective drugs in EGFR-WT NSCLC patients. In this study, we clustered functional modules related to the survival traits of EGFR-WT patients using weighted gene co-expression network analysis (WGCNA). We used these data to establish a two-gene prognostic signature based on the expression of CYP11B1 and DNALI1 by combining the least absolute shrinkage and selection operator (LASSO) algorithms and Cox proportional hazards regression analysis. Following the calculation of risk score (RS) based on the two-gene signature, patients with high RSs showed a worse prognosis. We further explored targeted drugs that could be effective in patients with a high RS by the connectivity map (CMap). Surprisingly, multiple HDAC inhibitors (HDACis) such as trichostatin A (TSA) and vorinostat (SAHA) that may have efficacy were identified. Also, we proved that HDACis could inhibit the proliferation and metastasis of NSCLC cells *in vitro*. Taken together, our study identified prognostic biomarkers for patients with EGFR-WT NSCLC and confirmed a novel potential role for HDACis in the clinical management of EGFR-WT patients.

## Introduction

Lung cancer has the highest morbidity and mortality in China and around the world. Most patients presented with lung cancer at a late stage owing to hidden onset and unspecific symptoms associated with the disease (1, 2). Lung cancer is generally classified into non-small-cell lung cancer (NSCLC) and small cell lung cancer (SCLC). However, this traditional classification according to histological assessment fails to account for the complex prognosis and drug resistance associated with the disease (3).

Radiotherapy combined with chemotherapy is the major treatment strategy for SCLC, whereas targeted therapy has become the first-line treatment for NSCLC patients carrying specific driver mutations (4–6). Epidermal growth factor receptor (EGFR)-tyrosine kinase inhibitors (TKI) such as gefitinib and erlotinib were the first targeted therapy for NSCLC. They have been widely applied in the clinical application for NSCLC patients carrying EGFR-sensitive mutations such as in-frame deletions at exon 19 and exon 21 point mutations (L858R). Also, EGFR-TKIs have significantly prolonged disease-free survival (DFS) compared with platinum-based chemotherapy (7, 8). However, only 20–30% of all NSCLC patients with EGFR-sensitive mutations can benefit from EGFR-TKIs. For patients with no EGFR gene mutations or an unknown mutation status, platinum-based doublet chemotherapy regimens remain the standard first-line therapy (9, 10). In these cases, the tumor response rate is estimated to be less than 10% and overall survival (OS) is only slightly improved (11). There is an unmet need to develop a novel therapy and to improve the prognosis for patients with EGFR wild-type (EGFR-WT) NSCLC.

The rapid development of bioinformatics analysis has allowed the development of novel biomarkers that can predict prognosis in patients with lung cancer (such as PD-L1 (12, 13), GLUT1 (14), and Ki-67 (15, 16)). However, little effort has been focused on the identification of specific biomarkers for EGFR-WT patients. Thioredoxin reductases 1 (TrxR1) has been reported to be related to the poor prognosis in EGFR-WT and ALK-negative NSCLC (17). As the statistical power of individual biomarkers is considered to be weak, it is necessary to establish a gene signature biomarker to improve the accuracy of prognosis prediction (18–20). Weighted gene co-expression network analysis (WGCNA) is a systems biology approach that clusters genes with a high co-expression relationship into the same module (21). WGCNA has been widely used to assess the functions of transcriptome systems (22), to identify gene modules related to clinical parameters and to investigate cancer biomarkers (23–25). However, WGCNA has not yet been reported to reveal the prognostic prediction of biomarkers in EGFR-WT NSCLC patients.

In this study, WGCNA was conducted on the expression profiles of EGFR-WT NSCLC patients and a two-gene prognosis signature was obtained by LASSO COX regression. We performed connectivity map (CMap) database analysis to identify HDACis as potential drugs to effectively target EGFR-WT NSCLC patients with a high risk score (RS). Our findings provided a further understanding for prognosis prediction and clinical treatment of EGFR-WT NSCLC patients.

## Materials and Methods

**Figure 1:**
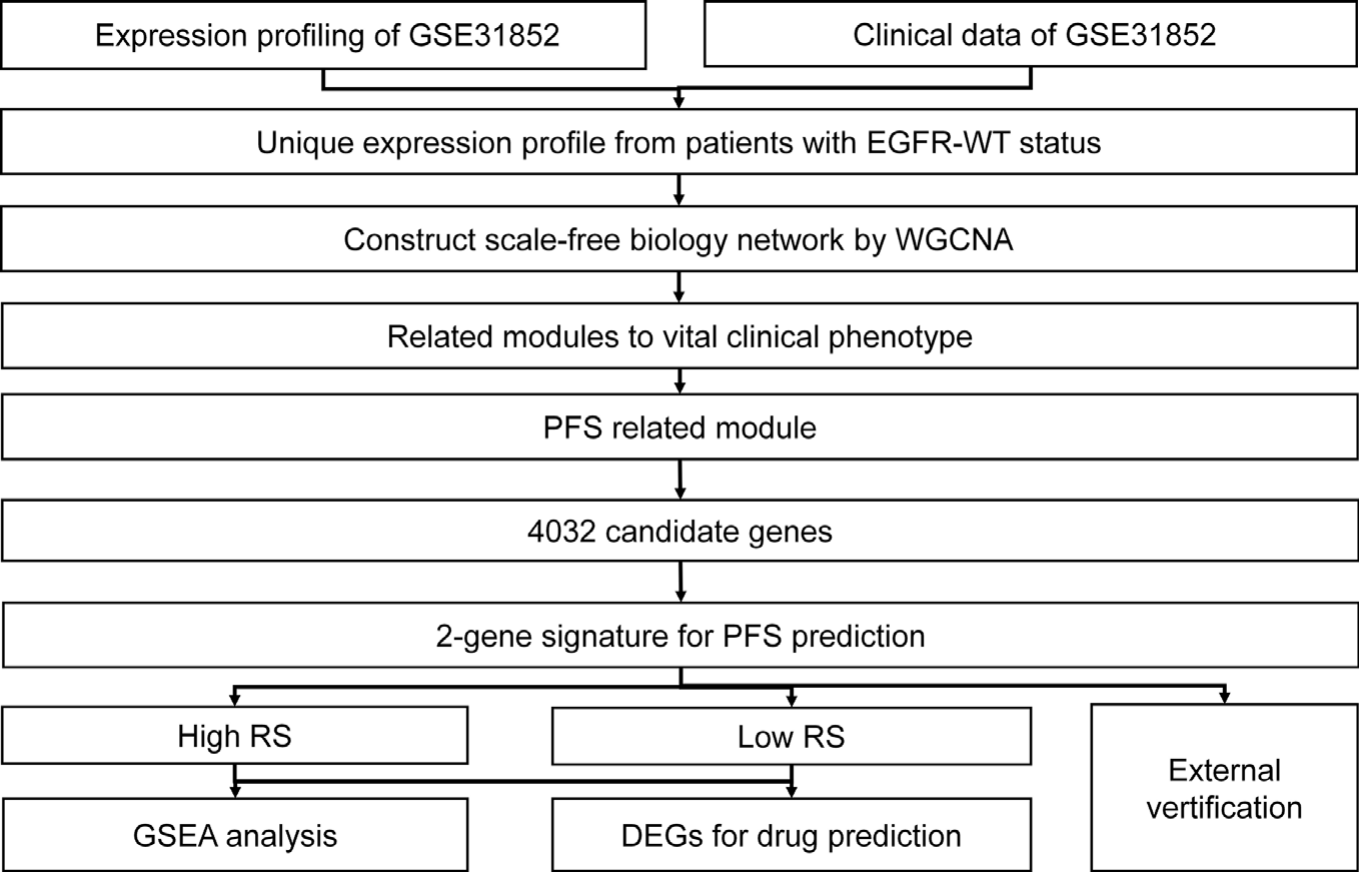
Schematic diagram of the bioinformatics process used for the analysis in this study.

The flow chart for this research is shown in **Figure 1**.

### Data Acquisition and Consolidation

GSE31852 database expression profile and clinical data were downloaded from the GEO database (**Table S1** ) as a training set, and 62 EGFR-WT patients with complete survival data were selected for further analysis (**Table S2**). Gene expression profiles of these samples were annotated by using the Human Gene 1.0 ST Array (**Table S3**, Affymetrix, Santa Clara, CA) according to Affymetrix protocols (**Table S4**). Probes with no gene or duplicate-gene annotation were excluded.

GSE31210 database (**Tables S5**-**S8**) was selected as the validation set, which contains the expression profile information, gene mutation status, and progression-free survival (PFS) of 226 NSCLC expression profiles (127 EGFR mutations, 20 KRAS mutations, 11 EML4-ALK fusion mutations, and 68 EGFR/KRAS/ALK-WT cases). Samples of 423 EGFR-WT and 75 EGFR-mutant LUAD patients from The Cancer Genome Atlas Program were downloaded from the The Cancer Genome Atlas (TCGA) database (https://portal.gdc.cancer.gov/) as another validation dataset (**Table S9**).

### WGCNA Network Construction

R package “WGCNA” was used for the automatic construction of a co-expression network. Firstly, a hierarchical clustering analysis of the samples was undertaken to ensure that there is little difference between the samples in the GSE31852 dataset (**Figure S1A**). The co-expression similarity matrix of gene expression was defined according to the Pearson correlation coefficient. Following the selection of an appropriate soft threshold *β*, the unweighted co-expression similarity matrix was converted into a weighted adjacency matrix. Then, the topological overlap matrix (TOM) was constructed using the degree of dissimilarity between the nodes and the dissimilarity index was defined between the nodes (26). Finally, by using the dynamic tree-cutting algorithm, the TOM was modified and the network modules were initially identified by satisfying conditions such that the difference between these modules is less than 0.25, or the similarity exceeds 0.75 (**Table S10**).

R package “WGCNA” was used sequentially to visualize the constructed network module and elucidate the correlation between external information. Modules with a significance *P* < 0.05 in the correlation test were defined being related to the trait. All genes in modules related to prognosis (time and status) were included in the construction of a prognostic risk signature for EGFR-WT patients.

### LASSO Regression and Multivariate COX Regression

R packages “glmnet” and “survival” were used to perform COX regression analysis through the LASSO algorithm. Those parameters with non-zero regression coefficients in the LASSO regression results were further included in the multivariate COX regression analysis. Genes with statistical significance in the multivariate Cox regression analysis were used to calculate their weighted gene expression values to establish RSs for each patient. The RS formula was established as follows:

$$\text{RS }=\text{ Ex}{\text{p}}_{\text{mRNA}1}\times \;{\text{β}}_{\text{mRNA}1}+\text{ Ex}{\text{p}}_{\text{mRNA}2}\times \;{\text{β}}_{\text{mRNA}2}+\;\ldots \;+\text{ Ex}{\text{p}}_{\text{mRNAn}}\times \;{\text{β}}_{\text{mRNAn}}$$

Exp_mRNA_ represents the expression level of each gene, and β_mRNA_ denotes the regression coefficient of the gene in the multi-factor COX regression model.

### Internal and External Verification of the Prognostic Risk Score Signature

X-tile software was used to calculate the best cut-off value of the patient’s RS. According to the best cut-off value, all patients were divided into a high-RS group and a low-RS group. Kaplan–Meier survival analysis was performed using the “survival” package with the “log-rank” method. Both the consistency parameter C-index of the survival model and the accuracy of the prediction model in the training set were validated by the resampling method for internal cross-validation using R package “boot.” R package “survivalROC” was used to plot the ROC curve and calculate the area under the curve (AUC).

### Gene Set Enrichment Analysis for Biological Function

GSEA Version 3.0 software was employed to enrich the main biological function pathways in the high-RS group, referring to “c2.cp.kegg.v6.2.symbols.gmt” and “h.all.v7.1.symbols.gmt” gene sets taken from the MsigDB database. All processes were performed according to the default parameters of the GSEA software. The number of random combinations was set to 1,000 and the results were sorted according to normalized enrichment scores (NES).

### Differential Gene Screening and Targeted Drug Prediction

Differentially expressed genes (DEGs) of the high-RS group *versus* low-RS group with |log fold change (log FC)| > 0.585 and *p*-value < 0.05 were analyzed by the R package “limma” (**Table S11**). Then, to find those drugs targeting high-RS patients, the differential gene sets were input into the CMap drug database (http://www.broadinstitute.org/cmap). The results included genes, diseases, or drug networks that were similar with, or opposite to, the expression profile. A positive score meant that the change in the expression profile caused by a drug was similar to the input gene expression profile. Conversely, a negative score indicated that the change in the expression profile caused by a drug was opposite that in the input gene expression profile. A drug with a negative score may reverse the corresponding gene expression in the disease and thus serves as a potential targeting drug for the disease (**Table S12**). Potential compound drugs were selected for verification according to the correlation score (less than 90) of the drugs (**Table S12**) based on published data from the literature (26).

### Cell Culture and Reagents

Lung adenocarcinoma cell lines A549 and H1299 without EGFR mutations were purchased from the Type Culture Collection of the Chinese Academy of Sciences (Shanghai, China), and cultured in RPMI-1640 (GibcoBRL, USA) supplemented with 10% fetal bovine serum (FBS), penicillin (100 U/ml), and streptomycin (100 mg/ml), in a humid atmosphere containing 5% CO_2_ at 37°C. Trichostatin A (TSA, HY-15144) and vorinostat (SAHA, HY-10221) were purchased from MedChem Express (Monmouth Junction, NJ, USA).

### MTT Assay

Approximately 2,000 cells per well in 96-well plates were treated with various concentrations of TSA or SAHA for 48 or 72 h. Then, we added 20 μl of 3-(4,5-dimethylthiazolyl-2-yl)-2,5-diphenyltetrazolium bromide (MTT) (5 mg/ml) to each well and the cells were incubated for another 4 h at 37°C. After removing the medium, cells were lysed in 200 ml dimenthylsulfoxide (DMSO) at room-temperature, and the optical density (OD) was measured at a wavelength of 570 nm with a microplate reader (Bio-Rad Laboratories, Hercules, CA, USA).

### Transwell Assay

Cells were treated with HDACis for 24 h, collected, and resuspend in serum-free media. Transwell chambers (Corning, NY, USA) were plated into a 24-well plate; 2 × 10^4^ cells in 200 μl of serum-free medium were seeded onto the upper chamber and 500 μl of medium with 10% FBS was added to the lower chamber with or without HDACis. After incubation for 24 h, the chambers were fixed with methanol and cells on the upper membrane were removed. Cells in the lower membrane were stained with Wright-Giemsa dye and the number of cells counted.

### Statistical Analysis

All statistical analyses and visualization were performed using GraphPad Prism 7.0 and R (version 3.6.2). Student’s *t*-test was used to compare the differences between the two groups: *P* < 0.05 was considered statistically significant.

## Results

### Data Collation and Patient Characteristics

In order to identify the key prognostic biomarkers for EGFR-WT NSCLC patients, we performed a systematic analysis of the GSE31852 dataset that included 62 patients with complete progression-free survival (PFS) information. The general information of these patients is summarized in **Table 1**. These patients included 24 lung adenocarcinomas, accounting for 38.7% of the samples, and six lung squamous cell carcinomas, accounting for 9.7% of the sample. The cohort consisted of 21 men and 16 women, accounting for 33.9 and 25.8% of the samples respectively. Six patients had no previous history of smoking, and 31 patients were former smokers. According to the expression profiles, all of the patients were clustered gene expression datasets (**Figure S1A**). As no obvious outliers in the expression profiles were observed, all of the 62 EGFR-WT patients were included in the subsequent analysis.

**Table 1:** Characteristics of patients of GSE31852.

Characteristic	Number (%)
Gender	
Male	21 (33.9)
Female	16 (25.8)
Unknown	25 (40.3)
Smoking status	
Never	6 (9.7)
Former	31 (50.0)
Unknown	25 (40.3)
Pathological stage	
I	1 (1.6)
II	2 (3.2)
III	7 (11.3)
IV	27 (43.5)
Unknown	25 (40.3)
Histology	
Adenocarcinoma	24 (38.7)
Squamous carcinoma	6 (9.7)
Unknown	32 (51.6)

### Construction of a Scale-Free Network by WGCNA

**Figure 2:**
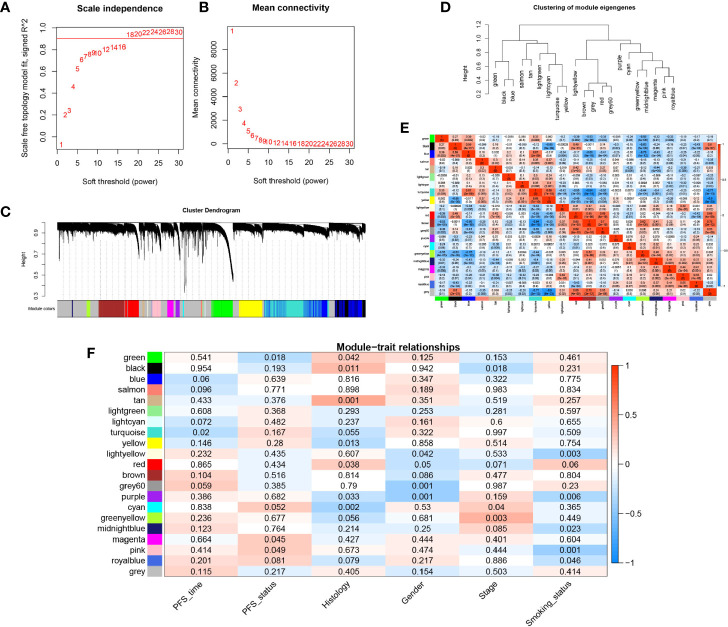
Construction of the weighted gene co-expression network analysis (WGCNA) network in EGFR-WT NSCLC patients and module identification. **(A)** Network topology for different soft-threshold powers. **(B)** The mean connectivity for different soft threshold powers. **(C)** One-step network construction by weighted gene co-expression network analysis (WGCNA). **(D)** Hierarchical clustering tree for clustering modules. **(E)** Heatmap of the correlation between identified modules **(F)** Correlation of modules and traits. Colors represent the correlation coefficient, while the number on each module represents the associated *P-*value.

WGCNA was conducted on the gene expression profile data to identify the gene network modules co-expressed in EGFR-WT patients and to explore the relationship between these gene network modules and prognosis. Firstly, a soft threshold (*β* = 18) with an *R* ^2^ > 0.9 was defined to establish an adjacency matrix (**Figures 2A, B**). All genes were clustered into 21 modules with different colors (**Figure 2C**). The gray module in which the genes were not clustered was excluded from the analysis. The similarity between each module was less than 0.75 (**Figures 2D, E**).

We calculated the module eigengenes (ME) value of the samples which represented the gene expression pattern of each module. We found that the gene expression of every sample in each module was different whereas the gene expression of each sample in every module was similar. These results suggested that the modules composed of genes with similar expression patterns may have important biological functions (**Figure S1B**). Then, according to gene significance (GS) which was defined as the association of a single gene with external information (clinical pathology parameters), the correlation between the survival parameters (PFS time and state) and related modules was explored (**Table S10**, **Figure 2F**). Among each of the 20 modules, the turquoise and green modules were both significantly associated with PFS (*P* < 0.05) compared to the other traits. These modules were most closely related to PFS time and PFS status with correlation coefficients of −0.42 (*P* < 0.0001) and −0.35 (*P* < 0.0001), respectively. These results indicated that the gene expression patterns in the turquoise and green modules were most closely correlated with prognosis in the EGFR-WT patients.

### Construction of Two-Gene Prognostic Signature-Based RS for EGFR-WT NSCLC Patients

**Figure 3:**
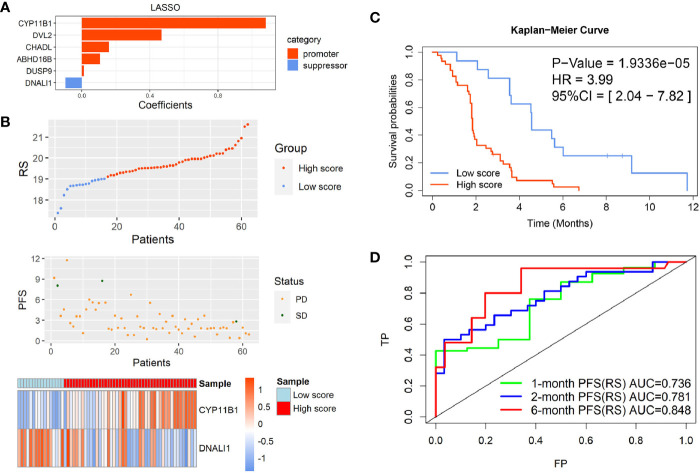
Identification of the two-gene prognostic signature-based risk score (RS) for EGFR-WT NSCLC patients. **(A)** Six robust markers obtained by LASSO regression. **(B)** The distribution of RSs in GSE31852. Top: two groups for EGFR-WT patients according to the best cut-off of risk score. Middle: relationship between RS (*X*-axis) and PFS time (*Y*-axis). Bottom: heatmap plot for the expression of genes in the two-gene signature. **(C)** Kaplan-Meier curve of PFS probability based on the RS in EGFR-WT NSCLC. **(D)** ROC curves were used to compare the predictive ability of the two-gene prognostic signature for 1, 2, and 6-month survival probabilities. PFS status: SD, stable disease; PD, progressive disease.

As each module in the WGCNA network based on gene expression profiles could be regarded as a characteristic for EGFR-WT patients, the turquoise module (**Figure S2A**) and green modules (**Figure S2B**) may contain the prognostic prediction genes for EGFR-WT patients. The genes of these two modules were combined as candidate gene sets for prognostic markers in EGFR-WT patients. To further select the key genes related to prognosis, we performed the Least Absolute Shrinkage and Selection Operator (LASSO) regression combined with multivariate COX regression analysis (**Figures 3A** and **S2C, D**). CYP11B1and DNALI1 were identified as independent prognostic factors for PFS of EGFR-WT NSCLC patients by univariate and multivariate Cox regression (**Table 2**). Based on the expression of independent prognostic factors and their corresponding coefficients in the regression analysis, the two-gene signature-based RS of each EGFR-WT patient was derived based on the following formula:

$$\text{RS }=\;\left[\text{Ex}{\text{p}}_{\left(\text{CYP}11\text{B}1\right)}\times \;3.3531005\right]\;+\;\left[\text{Ex}{\text{p}}_{\left(\text{DNALI}1\right)}\times \;\left(-0.6605697\right)\right]$$

**Table 2:** Univariate and multivariate Cox regression analysis between six candidate markers and progression-free survival (PFS) after Least Absolute Shrinkage and Selection Operator (LASSO) analysis.

Genes	Univariate COX				Multivariate COX			
	HR	95% CI	*P-*value		HR	95% CI	*P-*value	
ABHD16B	23.5951	3.5311–157.6667	0.00111	**	3.5896	0.4189–30.7607	0.2436	
CHADL	22.49484	5.13425–98.5573	0.00004	***	7.8693	0.6556–94.4554	0.1037	
CYP11B1	25.99976	5.63712–119.9172	0.00003	***	6.5785	1.2655–34.1969	0.0251	*
DNALI1	0.54419	0.33607–0.88118	0.01335	*	0.2759	0.1472–0.5174	0.0001	***
DUSP9	10.75214	3.09879–37.30756	0.00018	***	4.7842	0.6875–33.2923	0.1138	
DVL2	10.39295	2.73054–39.55755	0.0006	***	3.066	0.6193–15.1795	0.1698	

*P < 0.05，**P < 0.01，***P < 0.001.

According to the best cut-off value (RS = 19.1), the patients were divided into high- and low-RS groups (**Figure 3B**). Kaplan-Meier survival analysis showed that the PFS of patients in the high-RS group was significantly shorter than that in the low-RS group (**Figure 3C**, HR = 3.99, 95% CI = 2.04–7.82, *P* < 0.0001). The C-index was 0.8625 (95% CI = 0.7579–0.9671, *P* < 0.001) by internal cross-validation.

To assess the predictive ability of the model for short-term PFS, the receiver operating characteristic (ROC) curves of the two-gene signature-based on the RS and gene expression were drawn at 1, 2, and 6 months, and the AUC was determined. The results showed that the two-gene RS model was a good indicator of short-term PFS (**Figure 3D**). The AUCs for the two-gene RS at 1, 2, and 6 months were 0.736, 0.781, and 0.848, respectively. These values were significantly better than those obtained based on simple gene expression for predicting PFS at each time point (**Figures S2E, F**).

Taken together, these results indicated that the two-gene RS signature established by the PFS-related modules of WGCNA reflected the survival of EGFR-WT patients and had better predictive power than independent prognostic gene expression.

### External Validation of Two-Gene RS for EGFR-WT Patients

**Figure 4:**
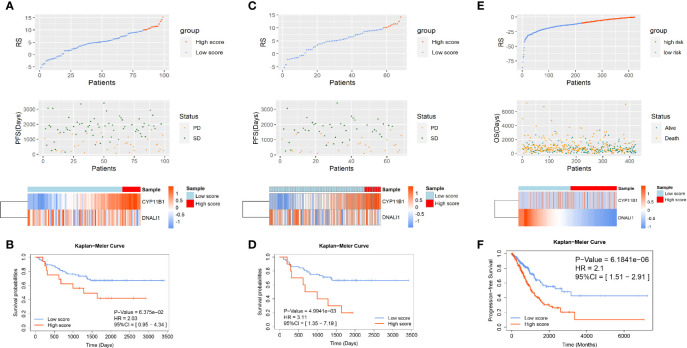
The verification of the two-gene prognostic signature for non-small-cell lung cancer (NSCLC) patients with EGFR-WT in GSE31210 and The Cancer Genome Atlas (TCGA) databases. The distribution of RSs in patients with **(A)** EGFR-WT, **(C)** EGFR/ALK/KRAS wild type in GSE31210, and **(E)** EGFR-WT in TCGA. Top: two groups for patients according to the best cut-off of RS. Middle: relationship between RS and PFS information. Bottom: heatmap plot for the expression of genes in the two-gene signature. Kaplan-Meier curve of PFS probability based on the RS in patients with **(B)** EGFR-WT, **(D)** EGFR/ALK/KRAS wild type in GSE31210, and **(F)** EGFR-WT in TCGA.

To validate the significance of the two-gene signature for the prognosis of PFS in EGFR-WT NSCLC patients, the GSE31210 database was selected for external verification. All patients were divided into high- or low-RS groups according to the best cut-off value (RS = 10.1) and analyzed based on the Kaplan-Meier survival curves. In patients with EGFR mutations, the PFS of low-RS group was significantly shorter than that of high-RS group (**Figures S3A, B**). In contrast, in patients with EGFR-WT (including KRAS mutations, ALK fusion, and none of three mutations named as triple-negative type), the PFS of high-RS patients tended to be shorter although it failed to reach statistical significance (*P* > 0.05) potentially due to the small sample size (**Figures 4A, B**). Further refinement of the grouping indicated that the two-gene RS signature had better performance in predicting PFS for patients with triple-negative disease (HR = 3.11, 95% CI = 1.35–7.19, *P* < 0.01) (**Figures 4C, D**). The C-index results of the prognostic model in the three classifications of populations also showed that the RS could fit the true situation of PFS for patients with triple-negative lung cancer (**Table 3**). However, no significant difference in the long-term OS was found between the high- and low-RS groups regardless of the types of mutations (**Figure S4**).

**Table 3:** C-index of the two-gene risk score (RS) signature in three different mutation populations.

Mutant	C-index	95% CI	*P*-value
EGFR-mutant	0.7051	0.4933–0.9169	0.0578
EGFR-WT	0.6600	0.4933–0.8268	0.0599
EGFR/KRAS/ALK-WT	0.7330	0.5887–0.8774	0.0016

Our results demonstrated that the two-gene RS was significant for survival prediction in EGFR-WT patients particularly in those who did not have EGFR/KRAS/ALK mutations. The two-gene signature was also verified in TCGA database (**Figures S3C, D** and **4E, F**). All NSCLC patients were also divided into high- and low-RS groups and analyzed from the Kaplan-Meier survival curves. The survival time of high-RS patients was significantly shorter than that of low-RS patients (HR = 2.1, 95% CI = 1.51–2.91, *P* < 0.0001), suggesting that the two-gene signature had a generalized and adaptive capacity in predicting the prognosis of EGFR-WT NSCLC patients.

### Verification of HDAC Inhibitors as Potential Targeted Drugs for EGFR-WT NSCLC

**Figure 5:**
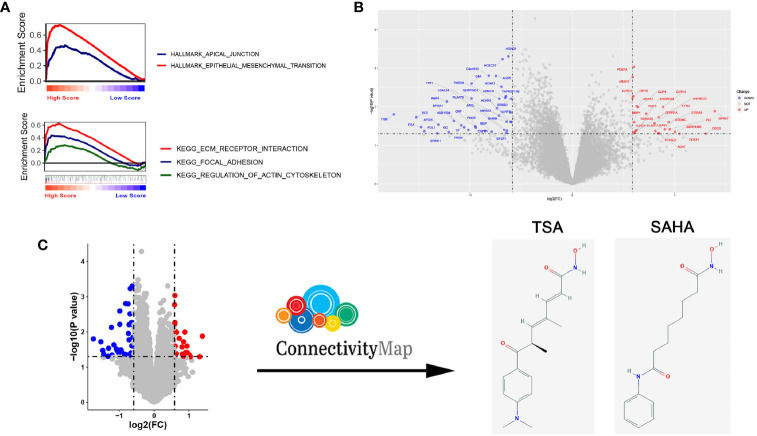
Compounds predicted to target EGFR-WT non-small-cell lung cancer (NSCLC) patients with a high risk score (RS) and verification *in vitro*. **(A)** Gene Set Enrichment Analysis (GSEA) of EGFR-WT NSCLC patients with high RS according to Kyoto Encyclopedia of Genes and Genomes (KEGG) pathways (top) and HALLMARK pathways (bottom). **(B)** Heatmap for the analysis of differentially expressed mRNA. **(C)** Schematic illustration for of the drug screening procedure through querying of the connectivity map (CMap).

To clarify the biological characteristics of high-RS populations, we performed GSEA on the gene expression profile of EGFR-WT NSCLC patients using the tumor-related HALLMARK pathway gene set and the classic Kyoto Encyclopedia of Genes and Genomes (KEGG) pathway gene set. Our results showed that both pathways were enriched in metastasis-related pathways including epithelial-mesenchymal transition, apical junction, focal adhesion, ECM receptor interaction and regulation of actin cytoskeleton. These data indicated that the poor prognosis of high-RS patients may be attributed to the enhancement of tumor metastatic ability (**Figure 5A**).

**Figure 6:**
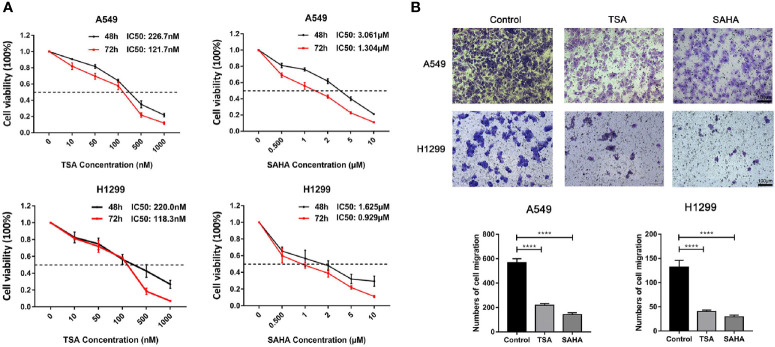
Verification of the effect of HDACis on EGFR-WT non-small-cell lung cancer (NSCLC) cells *in vitro*. **(A)** Inhibition of cell proliferation of trichostatin A (TSA) and vorinostat (SAHA) as assessed by MTT assay in A549 and H1299 cells. **(B)** Inhibition of migration ability by TSA and SAHA as assessed by Transwell assays in A549 and H1299 cells. ****P < 0.0001.

To ascertain potential drug targeting in the EGFR-WT high-RS population, we performed differential gene analysis between the high- and low-RS groups, and identified 25 up-regulated differential expression genes (DEGs) and 36 down-regulated DEGs (**Table S11**, **Figure 5B**). We compared these DEGs with the CMap database, aiming to identify the drugs that interacted with the DEGs as detailed in the *Materials and Methods* (**Figure 5C**). Twenty-four candidate drugs were identified, and interestingly, nine were classified as HDACi (**Tables 4**, **S12**). Considering the safety and feasibility of HDCAis for clinical applications, we verified two common HDACis, TSA, and SAHA in EGFR-WT lung cancer cells (A549 and H1299). Both HDACis significantly inhibited the proliferation and migration of these cell models (**Figures 6A, B**), indicating the potential of TSA and SAHA as novel treatment strategies in EGFR-WT lung cancer.

**Table 4:** Candidate drugs targeting EGFR-WT patients with high risk score (RS).

No.	Score	Name	Description
1	−98.94	Tracazolate	GABA receptor modulator
2	−95.3	SB-202190	p38 MAPK inhibitor
3	−95.14	Vemurafenib	RAF inhibitor
4	−94.88	THM-I-94	HDAC inhibitor
5	−94.76	Trichostatin-a	HDAC inhibitor
6	−94.58	NCH-51	HDAC inhibitor
7	−93.9	ISOX	HDAC inhibitor
8	−93.89	AZD-7762	CHK inhibitor
9	−93.84	TG-101348	FLT3 inhibitor
10	−93.81	HG-5-113-01	Protein kinase inhibitor
11	−93.62	Vorinostat	HDAC inhibitor
12	−93.59	Droxinostat	HDAC inhibitor
13	−93.16	SB-590885	RAF inhibitor
14	−93.09	Dasatinib	BCR-ABL kinase inhibitor
15	−92.74	Scriptaid	HDAC inhibitor
16	−92.61	HC-toxin	HDAC inhibitor
17	−92.39	Fostamatinib	SYK inhibitor
18	−92.11	KU-0063794	MTOR inhibitor
19	−91.53	TPCA-1	IKK inhibitor
20	−91.21	TWS-119	Glycogen synthase kinase inhibitor
21	−90.68	GSK-1070916	Aurora kinase inhibitor
22	−90.64	Pyroxamide	HDAC inhibitor
23	−90.41	PI-828	PI3K inhibitor
24	−90.13	PP-2	SRC inhibitor

### Discussion

In the current study, we established a RS based on a two-gene signature for EGFR-WT NSCLC patients and found that EGFR-WT patients with a high RS had a worse prognosis. Mechanistically, a high RS might cause poor prognosis by activating multiple metastasis-related pathways such as epithelial-mesenchymal transition and ECM receptor interaction. Furthermore, HDACis were screened out as potential targeted drugs for EGFR-WT NSCLC and found to inhibit cell proliferation and migration of EGFR-WT NSCLC cells.

Although well-known driver genes such as EGFR and ALK with sensitive mutations could strongly predict the efficacy of targeted drugs in NSCLC patients, about 50% of NSCLC patients without driver gene mutations do not benefit from the personalized targeted therapy (27, 28). For those patients that do not have corresponding mutations or have unknown mutation status, platinum-based doublet chemotherapy remains the standard first-line regimen which has poor efficacy (9, 10). In these patients, docetaxel or pemetrexed could be used as second-line single-agent chemotherapies. However, the tumor response rate was less than 10% and the OS was only slightly improved with these treatments (11). The current known tumor-driver mutations are not sufficient to fully predict the drug response of lung cancer. This study aimed to ascertain the characteristics of gene expression profiles in EGFR-WT NSCLC patients and provide evidence for the clinical treatment for NSCLC patients without specific mutations.

The rapid development of NGS technology and bioinformatics has allowed major progress in the prediction of NSCLC prognosis. In addition to the TNM stage, multi-gene prognostic signatures based on transcriptome sequencing have been developed for prognosis. However, the majority of studies of gene signatures for predicting prognosis in lung cancer have focused on RSs related to specific mechanisms, starting with functional molecules or cell components, such as immune infiltration (29–31), EMT scores (32), hypoxic or metabolic catabolites (33, 34), and non-coding RNA (35, 36). Little research has considered specific mutations.

Few gene signature studies have considered a scale-free property of the gene interaction network using WGCNA (37). WGCNA bridges the gap from individual genes to systems biology networks and can be utilized to identify hub genes that play key roles in the disease by converting the relationship between genes from a constant probability to the value adding a correlation weight (38). This allows the conversion from a random network to a scale-free network (39). Unfortunately, WGCNA on EGFR-WT NSCLC patients has not yet been reported.

In the present study, we used WGCNA to cluster the gene expression pattern of the EGFR-WT population, studied the co-expressed gene modules and correlated the gene modules with patient prognostic information for the first time. Due to the limited omics data containing information on mutations, the accuracy and comprehensiveness of our two-gene signature need to be validated in a large cohort of lung cancer patients. Also, we observed that the two-gene signature predicted the prognosis of patients without known driver gene mutations (EGFR/KRAS/ALK-WT) better than that of patients with EGFR-WT alone. Thus, the accumulation of next-generation sequencing (NGS) data in clinical tumor assessment and treatment may allow the further verification of a two-gene signature in a larger multi-omics database.

Considering the genes in WGCNA modules might be interrelated, they were not suitable for direct prognostic signature construction as this may result in multi-collinearity. We performed LASSO regression on the genes in the WGCNA modules to screen for the prognostic factors for the subsequent multivariate COX regression model. These analyses led to the selection of CYP11B1 and DNALI1 genes to construct a prognostic risk signature for EGFR-WT patients.

CYP11B1 encodes the cytochrome P450 family 11 subfamily B member 1 protein that is mostly expressed in the adrenal glands and is related to excessive cortisol secretion (40). In cancer, CYP11B1 is not only related to aldosterone- and cortisol-co-secreting adrenal tumors (41, 42), but it also affects the drug response of breast cancer (43), gastrointestinal tumors (44), leukemia (45), and other tumors. DNALI1 encodes dynein axonemal light intermediate chain 1 protein which is a human homologue of p28 in Chlamydia. DNALI1 is widely expressed in the human testis, ovaries and other tissues (28, 46), but the functions of DNALI1 in physiological processes and tumor development remain unclear. Limited studies have reported that DNALI1 is down-regulated in breast cancer (47) and negatively correlated with poor prognosis (48, 49). However, the expression pattern and function of both genes in lung cancer, as well as their influence on prognosis are unknown.

In this study, we analyzed the predictive significance of both molecules in the prognosis of NSCLC patients from online databases (**Figure S5**). The prognosis of patients with high expression of CYP11B1 or low expression of DNALI1 was significantly poor (*P* < 0.0001). Furthermore, the efficiency of prognostic prediction in the EGFR-WT population from RS based on the two-gene signature was better than that by individual genes (**Figures S2E, F**). The RS based on the two-gene signature had good predictive significance for the prognosis of EGFR-WT patients. This may provide a good reference value for clinical decision-making in EGFR-WT patients, particularly in patients without known driver mutations.

From the online drug database named CMap, HDACis were screened out as potential drugs to improve the prognosis of EGFR-WT patients with a high RS based on the two-gene signature.

CMap is a database developed by Broad Research Institute to reveal the functional relationships between small molecule compounds, genes, and disease states (50). CMap was employed to compare the differentially expressed gene list with the reference gene sets after specific treatments in the database. A correlation score (−100 to 100) was obtained according to the enrichment of differentially expressed genes in the reference gene expression profile. A positive score indicated that the up-regulation or down-regulation pattern of input genes was similar to the pattern of reference gene expression treated with different drugs. In contrast, a negative score indicated that the drugs regulated the expression of genes in an opposite direction. Finally, all treatments in the database were ranked according to the correlation score with the reference gene expression profile.

We found that among a total of 24 drug candidates with scores less than −90, 9 were HDACis, accounting for more than one-third of those identified. HDACis, such as romidepsin and vorinostat, are approved by the Food and Drug Administration (FDA) in cutaneous T cell lymphoma therapy (51, 52). However, although multiple preclinical studies have shown that HDAC inhibitors play a significant anti-cancer role *in vitro* or in animal models (53, 54), HDACis monotherapy clinical trials for lung cancer have failed (55–57). The results in the present study independently predicted that HDACis may be potential drugs for patients with high-RS EGFR-WT NSCLC and implicated the possibility of HDACis as single drugs for lung cancer therapy. Furthermore, HDACis were found to inhibit the proliferation and migration capacity of EGFR-WT NSCLC cells which was consistent with previous studies. Our research provides support for the independent application of HDAC inhibitors in EGFR wild-type NSCLC. Large-scale clinical trials should be carried out to confirm the efficacy of HDACis in EGFR-WT NSCLC patients.

In conclusion, the current study showed that a two-gene signature could effectively predict the survival of EGFR-WT patients, especially in NSCLC patients without known gene mutations. Our data also indicate that HDACis, such as TSA or SAHA, might be potentially effective clinical drugs for high-RS EGFR-WT patients. This study may fill the gap in lung cancer data analysis on one-specific mutant population, highlights the need for differential analysis of different oncomutations in cancer and also provides clues for the clinical treatment of EGFR-WT NSCLC patients.

## Data Availability Statement

The original contributions presented in the study are included in the article/**Supplementary Material**. Further inquiries can be directed to the corresponding authors.

## Author Contributions

YW and CZ analyzed the data and drafted the manuscript and all figures. WL and DW helped interpreted the data. YCheng and YChen completed analysis of drug prediction. KH and YL completed the *in vitro* experiments. JQ edited language grammars and all tables. XC and XH designed the study and revised the manuscript. All authors contributed to the article and approved the submitted version.

## Funding

This study was supported by the National Natural Science Foundation of China (Grant No. 81972197;No. 81472193), the Key Research and Development Program of Liaoning Province (2018225060), the Natural Science Foundation of Liaoning Province (2019-ZD-777), the Technological Special Project of Liaoning Province of China (2019020176- JH1/103), the Science and Technology Plan Project of Liaoning Province (No. 2013225585), Science and Technology Plan Project of Shenyang City (19–112–4–099).

## Conflict of Interest

The authors declare that the research was conducted in the absence of any commercial or financial relationships that could be construed as a potential conflict of interest.
