# Supplementary material for: Bioinformatics-Based Identification of HDAC Inhibitors as Potential Drugs to Target EGFR Wild-Type Non-Small-Cell Lung Cancer
Source: Front Oncol. 2021 Mar 8;11:620154. doi: 10.3389/fonc.2021.620154 (PMC7982742; doi:10.3389/fonc.2021.620154)
Supplement: Supplementary file 1 [file DataSheet_1.docx]

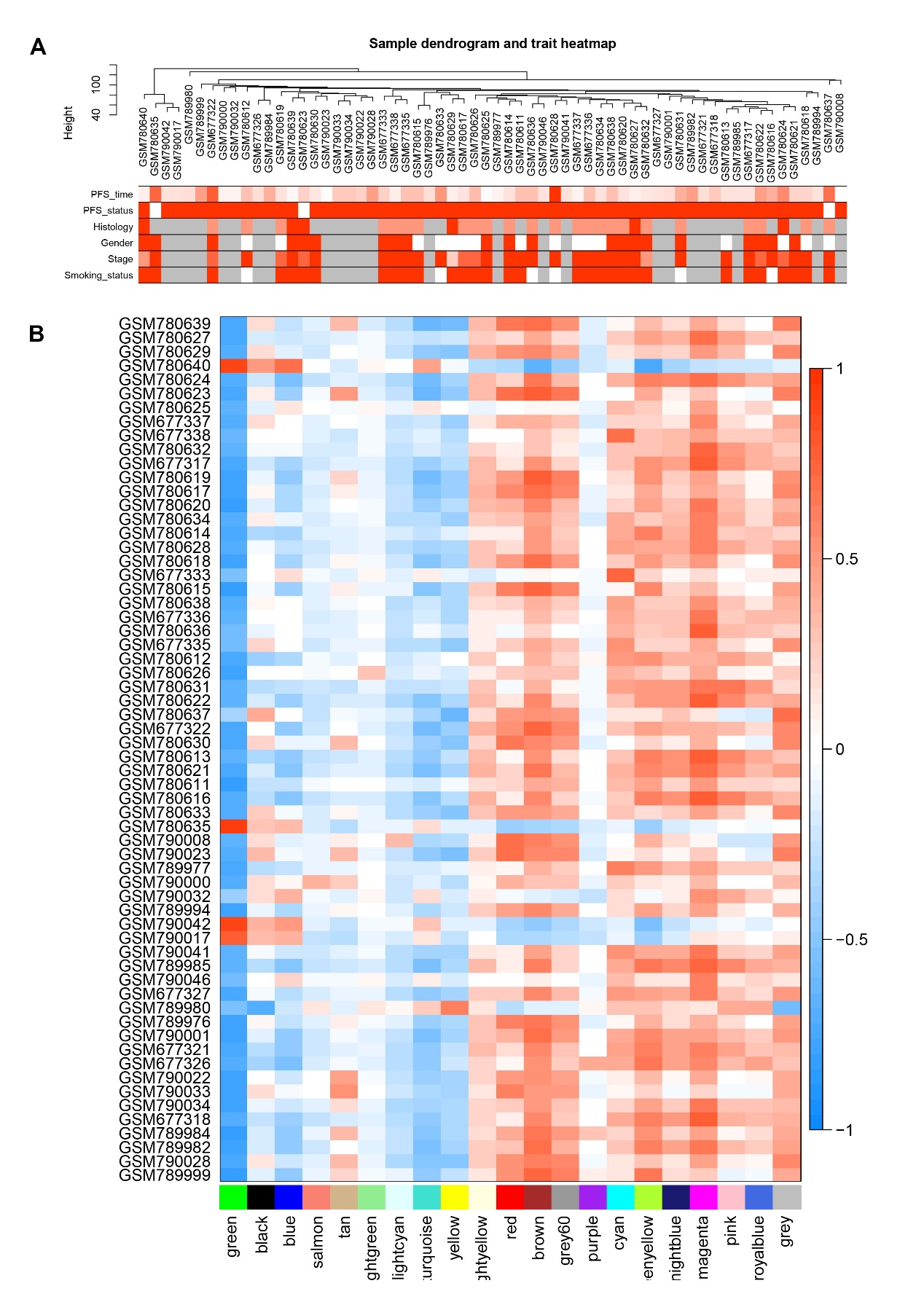


Figure S1 Characterizations of samples traits and gene modules in WGCNA.

(A) All samples included in GSE31852 and corresponding traits.

(B) Gene expression pattern of every sample in each module.


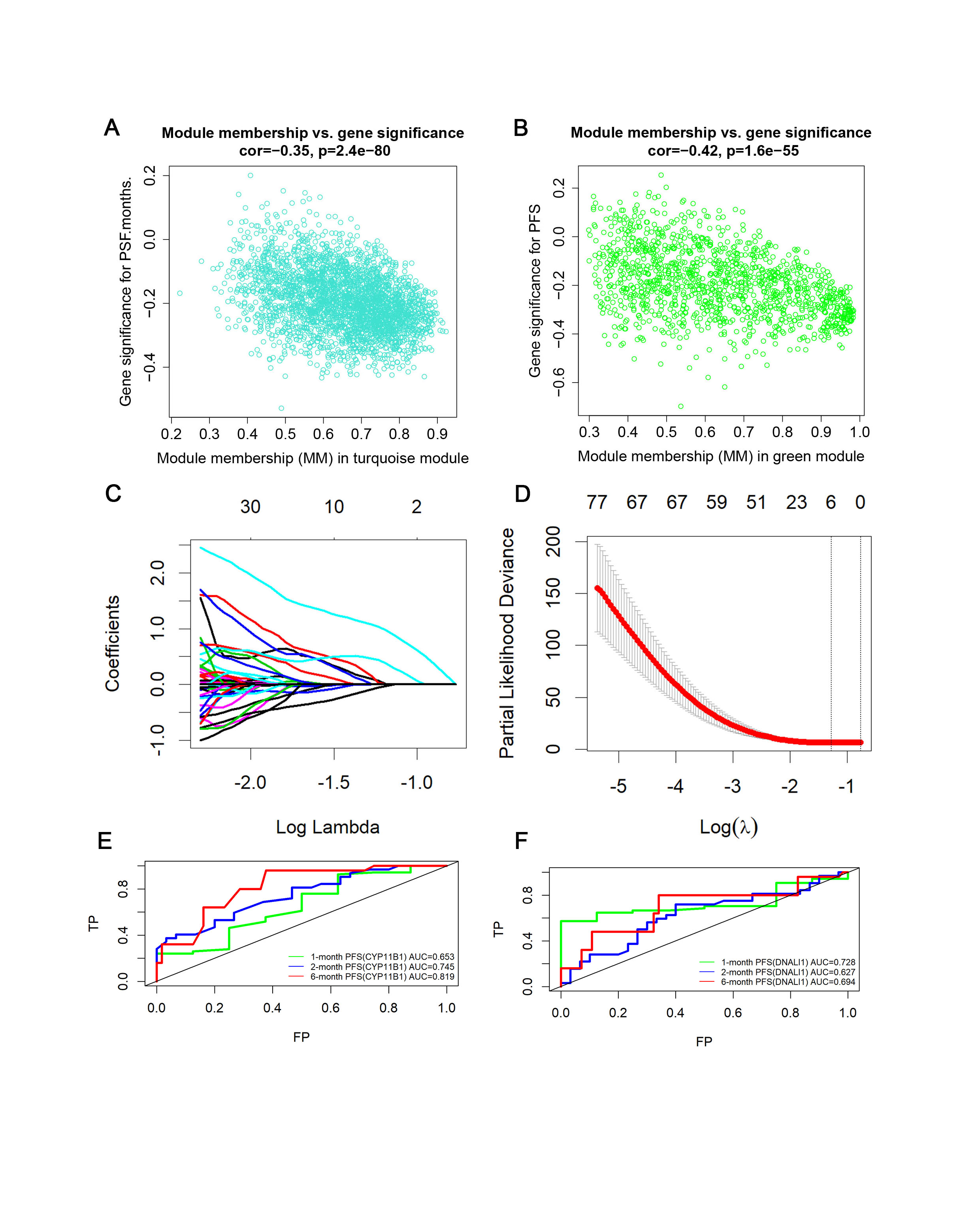


Figure S2 Construction of 2-gene prognostic signature for EGFR-WT NSCLC patients.

(A-B) the relationship of GS and MM for turquoise and green modules.

(C-D) Confidence interval for partial likelihood deviance as the lambda changed..

(E-F) ROC curves of each gene expression to predict survival independently.


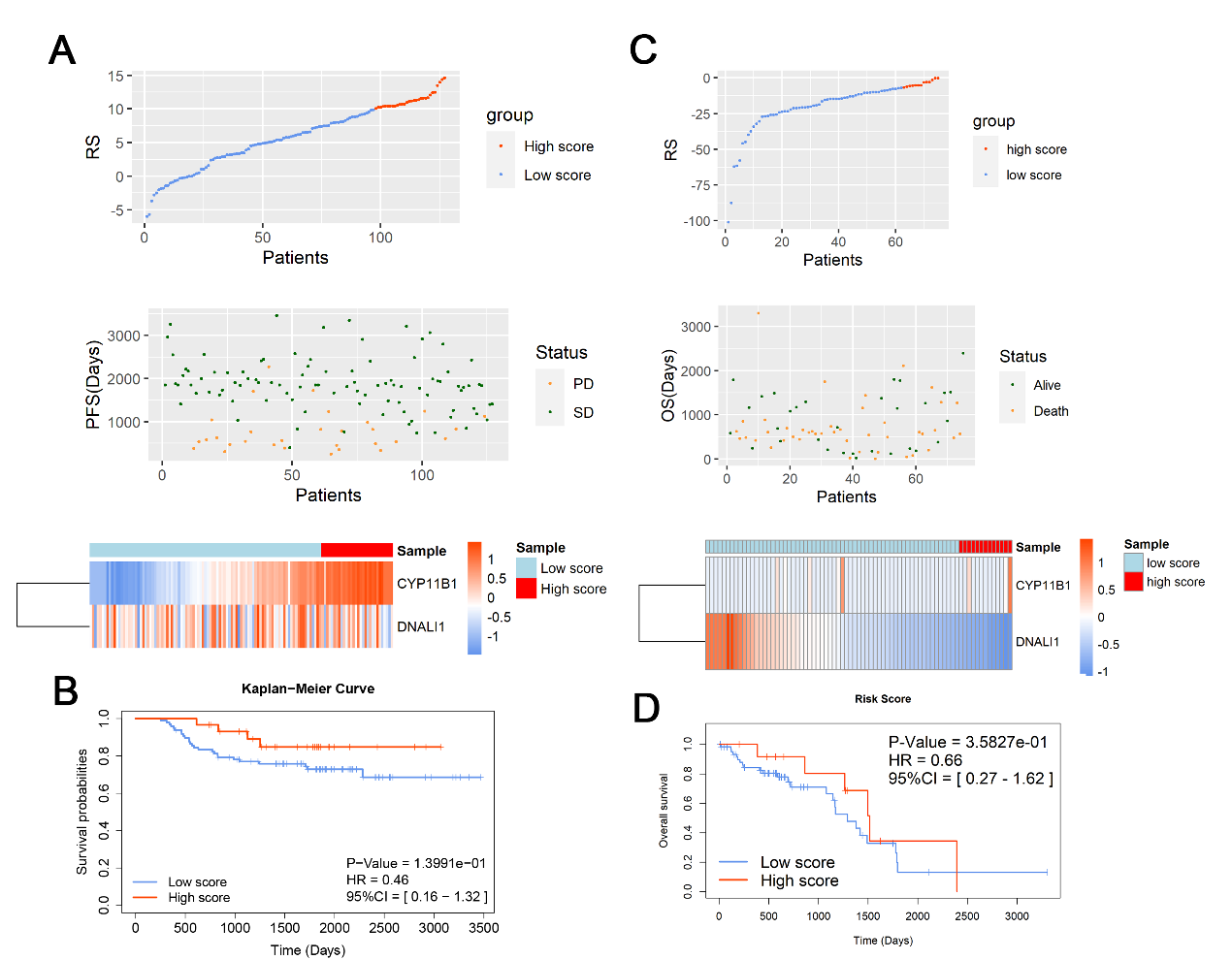


Figure S3. The verification of 2-gene prognostic signature for NSCLC patients EGFR sensitive mutation. The distribution of risk score in patients with EGFR sensitive mutation on PFS in GSE31210 (A) and on OS in TCGA (C). Top, two groups for patients according to the best cut-off of risk score. Middle, relationship between risk score and OS information. Bottom, heatmap plot for the expression of genes in the 2-gene signature. Kaplan-Meier curve of OS probability based on the RS in patients with EGFR sensitive mutation on PFS in GSE31210 (B) and on OS in TCGA (D).


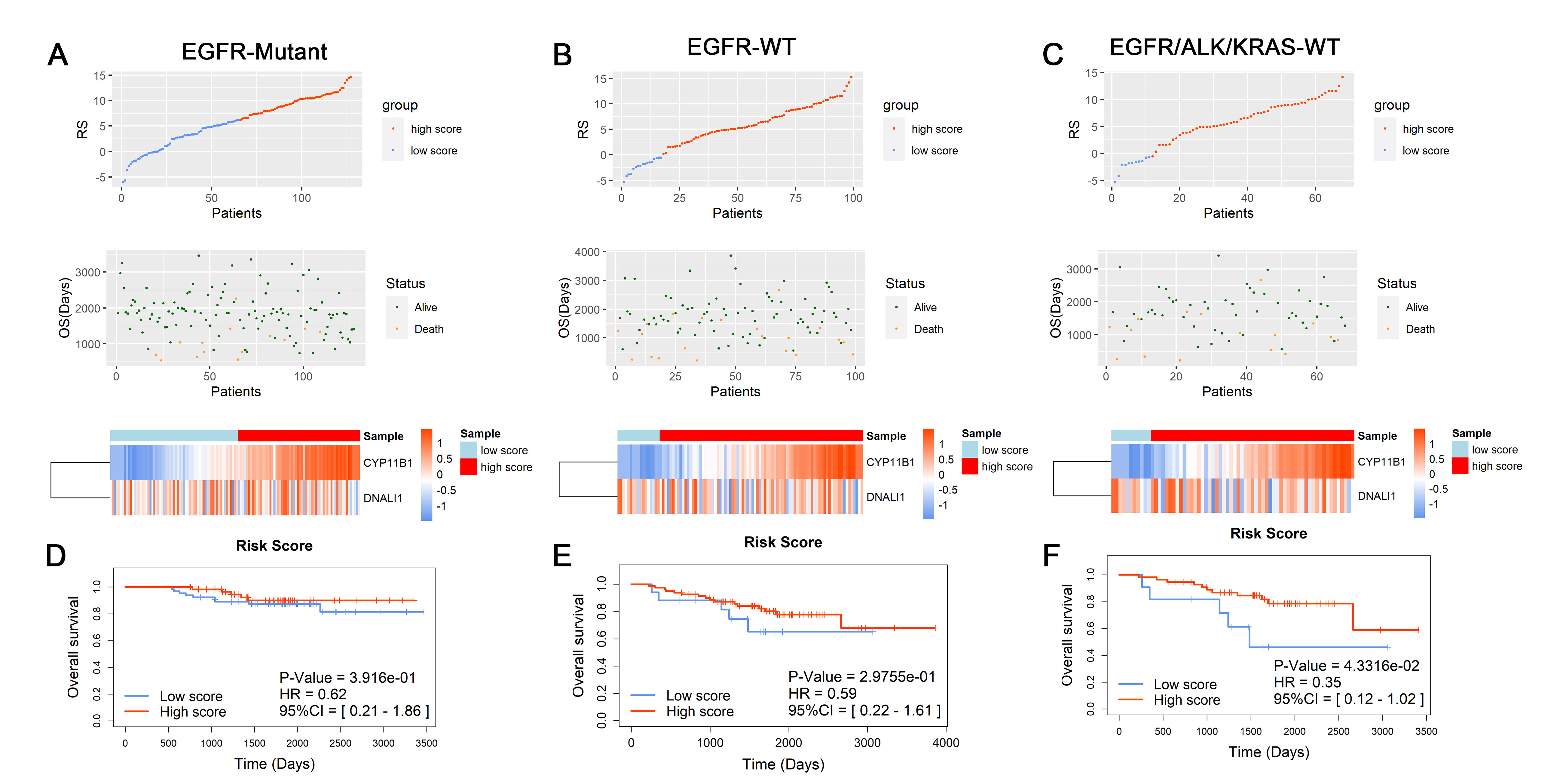


Figure S4. The verification of 2-gene prognostic signature for NSCLC patients with different mutation status on Overall survival (OS) in GSE31210. The distribution of risk score in patients with EGFR sensitive mutation (A), EGFR wild type (B) and EGFR/ALK/KRAS wild type (C). Top, two groups for patients according to the best cut-off of risk score. Middle, relationship between risk score and OS information. Bottom, heatmap plot for the expression of genes in the 2-gene signature. Kaplan-Meier curve of OS probability based on the RS in patients with EGFR sensitive mutation (D), EGFR wild type (E) and EGFR/ALK/KRAS wild type (F).


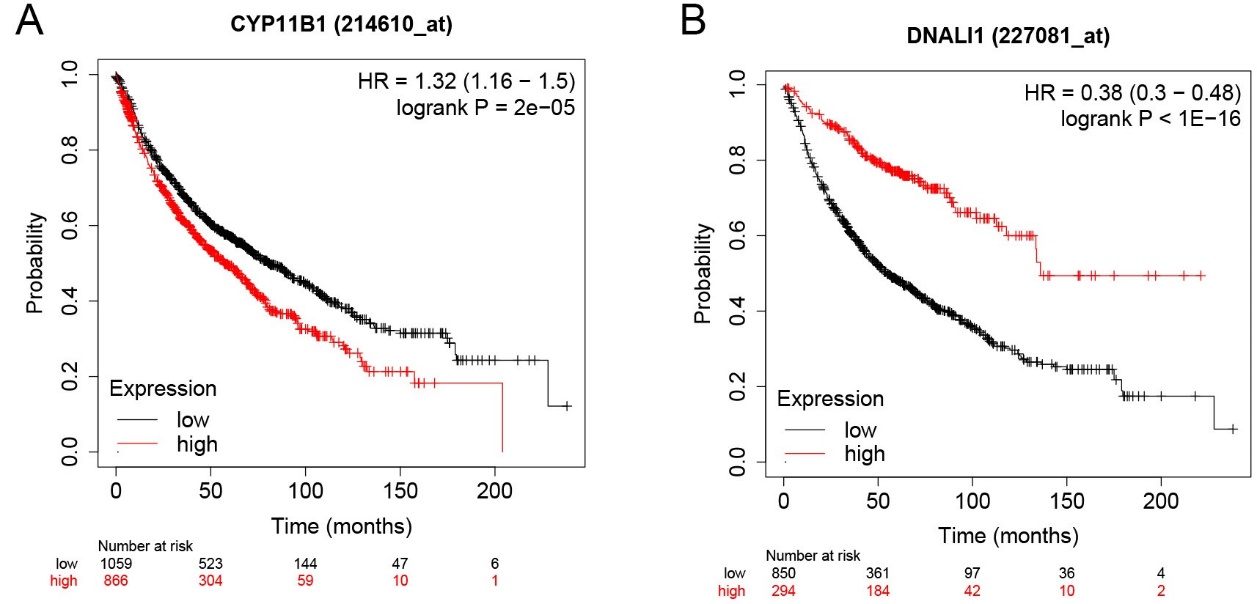


Figure S5. Prediction of survival of two gene in 2-gene prognostic signature separately. (A) CYP11B1, (B) DNALI1
